# External Fixation in the Treatment of Proximal Humeral Fractures: A Retrospective Single-Center Case Series

**DOI:** 10.3390/jcm15093432

**Published:** 2026-04-30

**Authors:** Gianfilippo Caggiari, Emanuele Ciurlia, Stefano Pescia, Alessandro Isola, Sebastiano Ortu, Andrea Donato, Edoardo Fantinato, Lucia Piras, Corrado Ciatti, Leonardo Puddu, Filippo Migliorini, Mario Manca, Carlo Doria

**Affiliations:** 1 Orthopaedic Department, Sassari University Hospital, Viale San Pietro 43b, 07100 Sassari, SS, Italy; luciapiras1997@gmail.com; 2 Orthopaedic Department, Ospedale Santissima Trinità di Borgomanero, Viale Zoppis 10, 28021 Borgomanero, NO, Italy; ciurlia81@gmail.com; 3 Orthopaedic Department, IRCCS University Hospital San Gerardo, Via G.B. Pergolesi 33, 20900 Monza, MB, Italy; stefano.pescia@gmail.com; 4 Orthopaedic Department, Versilia Hospital, Via Aurelia 335, 55041 Lido di Camaiore, LU, Italy; axlislasnd4@hotmail.com (A.I.); mario.manca@uslnordovest.toscana.it (M.M.); 5 Orthopaedics and Traumatology Department, Santissima Annunziata Hospital, AUSL Ferrara, Via Giovanni Vicini 2, 44042 Cento, FE, Italy; sebastiano.ortu10@gmail.com; 6 Orthopaedic Department, Mater Olbia Hospital, SS 125 Orientale Sarda, 07026 Olbia, SS, Italy; andonato88@gmail.com; 7 Orthopaedic Department, ASST dei Sette Laghi, Viale Borri 57, 21100 Varese, VA, Italy; edo.fantinato@gmail.com; 8 Orthopaedics and Traumatology Department, Guglielmo da Saliceto Hospital, University of Parma, AUSL Piacenza, 29121 Piacenza, PC, Italy; dadociatti@icloud.com; 9 Orthopaedic Department, Rovereto Hospital, Corso Verona 4, 38068 Rovereto, TN, Italy; l.puddu86@gmail.com; 10 Department of Trauma and Reconstructive Surgery, University Hospital of Halle, 06120 Halle, Germany; migliorini.md@gmail.com; 11 Department of Life Sciences, Health, and Health Professions, Link Campus University, 00165 Rome, RM, Italy; 12 Department of Orthopaedic and Trauma Surgery, Academic Hospital of Bolzano, 39100 Bolzano, BZ, Italy

**Keywords:** shoulder, external fixation, proximal humeral fractures, minimally invasive surgery

## Abstract

**Purpose**: The treatment of proximal humerus fractures (PHFs) remains debated, and similar fracture patterns may be managed with different strategies. The aim of this retrospective single-center case series without a control group was to evaluate clinical and radiographic outcomes after treatment of selected PHFs with the Galaxy Fixation System. The primary endpoint was functional recovery at 12 months, assessed using the Constant Shoulder Score and QuickDASH. Secondary endpoints included radiographic maintenance of reduction, quality of life, treatment-related complications, and need for revision surgery. **Methods**: We retrospectively analyzed 48 consecutive patients with proximal humeral fractures treated at the Orthopaedic and Traumatology Unit of Versilia Hospital, Viareggio, Italy, between November 2017 and February 2022. Fractures were assessed using trauma-series radiographs and computed tomography when required, and were classified by two senior surgeons according to the Neer, AO/OTA, and Hertel classifications. Eligible patterns included 2-part, 3-part, and selected 4-part fractures with at least two-thirds of intact metaphyseal bone stock. **Results**: Forty-six patients completed the 12-month follow-up; two patients died during follow-up from causes unrelated to the index procedure. The mean Constant Shoulder Score improved from 62.7 at 6 months to 69.3 at 12 months, and the mean QuickDASH improved from 9.4 to 8.1. The mean postoperative head-shaft angle was 137.2 degrees and remained substantially stable at 135.1 degrees at 12 months. Pin-tract infection occurred in 5 patients, pin migration in 4, algodystrophic syndrome in 1, and avascular necrosis requiring revision arthroplasty in 1. **Conclusions**: In this retrospective uncontrolled series, external fixation with the Galaxy system was associated with progressive functional recovery, satisfactory radiographic maintenance of reduction, and a low rate of revision surgery in carefully selected PHFs. These findings should be interpreted cautiously because of the retrospective design, limited sample size, absence of a control group, incomplete availability of some baseline variables, and lack of formal comparative or cost-effectiveness analyses.

## 1. Introduction

Proximal humerus fractures (PHFs) represent one of the most frequent fragility fractures encountered in daily orthopedic practice and remain a relevant source of disability, pain, and loss of independence, particularly in older adults [1]. After proximal femoral and distal radius fractures, PHFs are among the most prevalent fractures in the elderly population and currently account for approximately 5% of all adult fractures [2]. Their incidence has progressively increased over recent decades, mainly because of an aging population, longer life expectancy, and the growing prevalence of osteoporosis [3]. This epidemiological trend has important implications not only for surgeons but also for healthcare systems, rehabilitation services, and long-term care pathways [4].

The mechanisms of injury vary according to patient age and bone quality. In elderly individuals, PHFs most often follow low-energy trauma, especially accidental falls from standing height, and are frequently associated with osteoporotic bone and frailty-related comorbidities [5]. By contrast, in younger patients these fractures more commonly result from high-energy trauma, such as road traffic accidents, sports injuries, or work-related trauma. In these settings, the fracture may be associated with more extensive soft-tissue damage and a broader spectrum of fracture patterns, thereby increasing treatment complexity [6].

Despite their frequency, the management of proximal humerus fractures remains controversial. Therapeutic decision-making is influenced by multiple variables, including fracture morphology, displacement, integrity of the medial calcar, bone quality, patient age, baseline autonomy, functional requirements, comorbidities, and surgeon experience. As a consequence, similar fracture patterns may be treated with markedly different strategies in different institutions, and the literature still does not provide a universally accepted algorithm for every clinical scenario [7,8,9].

A further source of controversy is the imperfect reproducibility of the most commonly used classification systems. Although Neer, AO, and Hertel classifications are widely adopted, interobserver agreement is often only moderate, especially in multifragmentary fractures or when radiographs are suboptimal. This means that treatment indication is rarely based on classification alone and instead emerges from an integrated assessment of displacement, metaphyseal support, bone quality, and patient-specific functional goals. In practical terms, surgeons are often required to adapt the treatment plan to a combination of anatomical and clinical considerations rather than to a single classification label.

Treatment options range from conservative management to several operative techniques. Non-operative treatment remains appropriate for stable or minimally displaced fractures, particularly in frail or low-demand patients. Surgical management is generally reserved for displaced, unstable, or multi-fragmentary patterns and may include percutaneous pinning, intramedullary nailing, locking plate fixation, hemiarthroplasty, reverse shoulder arthroplasty, or external fixation. Each solution has specific advantages and limitations, and no technique is free from complications. In particular, osteoporotic bone, tuberosity displacement, metaphyseal comminution, and loss of medial support are well-known risk factors for fixation failure, varus collapse, or unsatisfactory functional recovery.

The growing interest in joint-preserving surgery has also shifted attention toward techniques that maximize biology and minimize soft-tissue aggression. In the proximal humerus, this concept is particularly relevant because the blood supply of the humeral head can be jeopardized both by the fracture itself and by extensive surgical exposure. Consequently, a minimally invasive technique that permits acceptable alignment without large deltopectoral dissection may offer theoretical biological advantages, particularly in borderline cases in which fixation is possible but excessive surgical trauma is undesirable.

Within this complex scenario, minimally invasive external fixation has gained renewed interest as a biologically respectful strategy for selected PHF. The Galaxy Fixation System™ can be used in damage control settings, as a definitive osteosynthesis device, and in reconstructive procedures. The system is versatile, allows percutaneous fixation with limited soft tissue stripping, preserves fracture biology and humeral head vascularity, and may be associated with a shorter learning curve than more demanding reconstructive procedures. Furthermore, the absence of retained internal hardware may simplify postoperative management and the treatment of eventual late complications.

The rationale for using external fixation in proximal humerus fractures lies in the possibility of combining minimally invasive reduction, respect for the fracture hematoma, and satisfactory mechanical stability. By avoiding an extensive surgical approach, this technique aims to reduce additional vascular insult to the humeral head while still allowing fixation of the proximal fragment and restoration of alignment. The modular configuration of the device may also permit minor postoperative adjustments during the healing phase, which is a distinctive advantage compared with other fixation methods.

Another relevant issue is the balance between radiographic perfection and functional sufficiency. In elderly or medically fragile patients, a treatment capable of providing pain control, restoration of acceptable alignment, and early mobilization may be preferable to a more invasive strategy aimed at anatomic reconstruction but burdened by longer operative times and higher implant-related risks. For this reason, the evaluation of external fixation should not be limited to radiographic parameters alone; its value should also be judged in relation to complication avoidance, postoperative manageability, and speed of functional recovery.

From an organizational standpoint, techniques with shorter learning curves and reproducible steps may also be advantageous in trauma systems with multiple surgeons sharing the same emergency workload. The external fixation system assessed in the present study was used by operators with different levels of shoulder-surgery specialization, making it possible to explore not only clinical outcome but also the practical transferability of the method in routine hospital activity.

However, the success of external fixation depends on careful patient selection, detailed preoperative planning, precise pin placement, and rigorous postoperative surveillance, especially with regard to pin-tract care and rehabilitation. For this reason, further clinical evidence is useful to define the role of this technique among the currently available treatment options.

The aim of the present study was therefore to evaluate clinical and radiographic outcomes at 6 and 12 months in a consecutive series of patients with proximal humerus fractures treated with the Galaxy Fixation System at a single trauma center [10]. The primary endpoint was functional recovery at 12 months, assessed using the Constant Shoulder Score and QuickDASH. Secondary endpoints included maintenance of reduction assessed by the head-shaft angle, SF-12 quality-of-life scores, treatment-related complications, and need for revision surgery.

## 2. Materials and Methods

We performed a retrospective single-center case series without a control group. The study included 48 consecutive patients with proximal humerus fractures who underwent surgical treatment with external fixation at the Orthopaedic and Traumatology Unit of Versilia Hospital, Viareggio, Italy, between November 2017 and February 2022.

The study population consisted of 36 women and 12 men, with a mean age of 68 years (range, 41–85 years). All patients were followed clinically and radiographically for at least 12 months after surgery. Standard preoperative imaging included anteroposterior and lateral trauma-series radiographs of the shoulder. Computed tomography was obtained whenever plain radiographs were considered insufficient to define fracture morphology, assess tuberosity displacement, or support surgical planning. Available baseline demographic, clinical, and fracture-related characteristics are summarized in Table 1. Variables requested for transparent reporting but not available in the retrospective database, including exact right/left laterality distribution, dominance, patient-level osteoporosis counts, and numerical distribution of fracture classes, are explicitly indicated as not reported.

All fractures were independently reviewed by two senior orthopedic surgeons and classified according to the Neer, AO/OTA, and Hertel classification systems [1,2,3]. The use of multiple classifications allowed a more detailed characterization of fracture morphology and helped identify patterns suitable for external fixation. Surgical indication included 2-part, 3-part, and selected 4-part proximal humerus fractures with at least two-thirds of intact metaphyseal bone stock [10]. Because of the retrospective nature of the dataset, the numerical distribution of fractures by Neer, AO/OTA, and Hertel categories was not available for reliable tabulation and is therefore reported as not available rather than reconstructed post hoc.

Exclusion criteria comprised ASA 4 status or severe systemic illness, multiple major comorbidities, active cancer or ongoing chemotherapy, chronic steroid therapy, sepsis, and local or systemic infection at the time of surgery. Patients in whom the fracture pattern was deemed unsuitable for stable external fixation, or in whom arthroplasty was considered more appropriate, were not included in this series. The total number of screened patients and the numerical distribution of exclusions by reason were not recorded in the available retrospective dataset; this limitation is explicitly reported in the patient flow summary.

The mean time from trauma to surgery was 1.35 days (range, 0–3 days). Early surgery was preferred whenever possible in order to reduce patient discomfort, facilitate reduction maneuvers, and start postoperative mobilization without unnecessary delay.

All procedures were performed by 11 surgeons working in the same department. In Section 3, the operators are described as five high-volume and six lower-volume shoulder surgeons. No formal learning-curve or inter-operator variability analysis was performed; therefore, any interpretation regarding reproducibility or transferability of the technique is presented only as an observational consideration.

### 2.1. Surgical Technique

**Figure 1:**
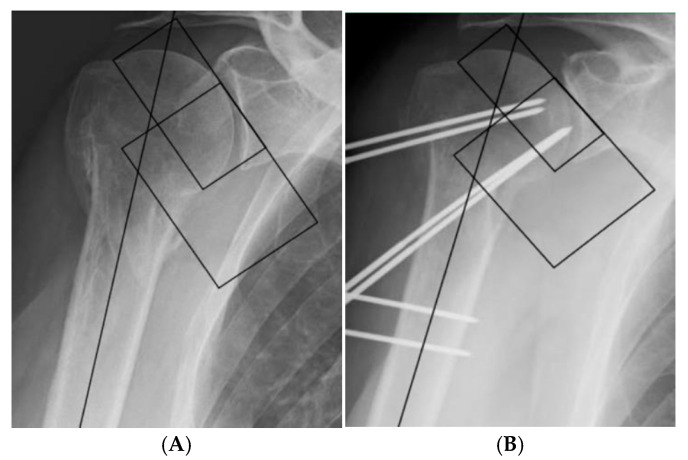
(**A**) Preoperative radiograph: the shoulder joint is positioned parallel to the body and perpendicular to the X-ray beam. The image is obtained with the C-arm rotated 45° posteriorly, with the beam directed through the center of the glenohumeral joint to optimize visualization of the surgical field and intraoperative anatomy. (**B**) Intraoperative radiograph after fixation with six fully threaded 2.5 mm pins to stabilize the proximal humerus.

Patients were positioned in the beach-chair position. The image intensifier was placed at the head of the bed and aligned with the longitudinal axis of the table, while the C-arm was rotated approximately 45 degrees anteriorly with the receiver positioned superiorly. The injured arm was kept parallel to the body and orthogonal to the X-ray beam directed through the center of the glenohumeral joint. This setup allowed acquisition of true anteroposterior views in neutral and rotational positions without repeated repositioning of the fluoroscopy unit (Figure 1A).

Preliminary fracture reduction was performed by axial traction along the humeral shaft with the arm in neutral rotation, followed by gentle abduction up to approximately 120 degrees until the proximal fragment aligned with the acromial profile. A retropulsion maneuver of the humeral shaft at 90 degrees of abduction completed the reduction [10]. Particular attention was paid to the restoration of medial calcar support, either by direct reduction or by slight medialization of the proximal fragment over the distal shaft, in order to decrease the risk of postoperative varus collapse. In the standard construct, six fully threaded 2.5 mm pins were used [10] (Figure 1B).

The first pin was inserted along the lateral humeral line, approximately 9 cm distal to the acromion and 1 cm anteriorly, with the trajectory directed toward the coracoid process while respecting humeral retroversion and crossing the fracture site. The second pin was placed parallel to the first along the same lateral line. A protective sleeve was used during pin insertion to reduce the risk of soft-tissue injury, especially to the axillary nerve. Pins were advanced up to the subchondral bone of the humeral head to achieve bicortical stabilization and rotational control [10,11,12,13].

A second pair of pins was then introduced proximal to the fracture line and directed toward the subchondral bone of the humeral head. The final two pins were positioned distal to the fracture line and perpendicular to the shaft, approximately at the same level as the initial pins [14,15,16,17,18]. The exact sequence of pin insertion could be modified according to the fracture pattern and surgeon preference. For example, in valgus-displaced humeral heads, the proximal pins could be used first as joysticks to achieve reduction before completion of the frame.

The medial convergence of four pins contributed to stabilization of the medial hinge, while the lateral arrangement of paired pins improved resistance to shear stress and opposed deforming forces generated by the rotator cuff. Pins were linked by locking clamps and connected to 6 mm rods through pin-to-rod and rod-to-rod connectors in a lambda configuration [10,19]. In fractures with marked tuberosity displacement, reduction could be facilitated through a mini-incision of the rotator cuff. No case required a formal open reduction. Antibiotic prophylaxis consisted of intravenous ceftriaxone 2 g; in allergic patients, vancomycin 1 g was administered.

### 2.2. Postoperative Care

Pins were dressed weekly until removal of the Galaxy system, which occurred after a mean of 43 days (SD ± 3.12). Postoperative rehabilitation was considered a key component of treatment and was tailored to pain, fracture configuration, and patient compliance. Early mobilization was encouraged in order to reduce stiffness and support recovery of daily activities, while still protecting the healing fracture.

Finger, wrist, and elbow mobilization was started immediately. Gentle passive glenohumeral mobilization was started after the first postoperative week according to pain tolerance. Pendulum exercises, particularly in forward flexion, were allowed from the second week together with cryotherapy and oral analgesics when required. Particular vigilance was maintained for signs of anterior bursitis due to acromial impingement from the pins, because untreated local inflammation could extend to the pin tracts and compromise fixation.

Between the third and sixth postoperative weeks, patients progressively started active glenohumeral motion, whereas scapulothoracic exercises were intensified. After frame removal, patients were encouraged to move the shoulder freely and to begin gradual rotator-cuff strengthening. The rehabilitation protocol aimed not only to restore range of motion, but also to support neuromuscular recovery, pain control, and a safe return to autonomy in activities of daily living.

The postoperative protocol deserves additional consideration because the benefits of a minimally invasive fixation can be lost if rehabilitation is either too aggressive or excessively delayed. A staged program that protects reduction during the first weeks while progressively restoring glenohumeral motion is essential. In frail older adults, early supervised movement may also reduce the global deconditioning associated with immobilization, including dependence in dressing, hygiene, and transfers. Therefore, the rehabilitation pathway should be viewed as an integral component of the treatment rather than a secondary phase occurring after surgery.

### 2.3. Follow-Up

Standard anteroposterior shoulder radiographs in neutral and rotational positions were obtained at every follow-up visit. Head-shaft angle was measured on true anteroposterior radiographs on the first postoperative day and again at frame removal or subsequent follow-up in order to assess maintenance of reduction and construct stability. Measurements were performed according to Adrikishna et al. [4,6] by two orthopedic surgeons using the standard imaging tools available in our picture archiving and communication system.

In addition to functional scores, radiographic maintenance of reduction remains clinically meaningful because progressive varus collapse is often associated with poorer shoulder mechanics, pain, and reduced leverage of the rotator cuff. The preservation of head-shaft angle observed in our cohort suggests that the construct was capable of resisting the deforming forces acting on the proximal fragment during healing. This aspect is particularly relevant in osteoporotic fractures, in which metaphyseal comminution and medial support deficiency frequently predispose to secondary displacement.

All collected data were entered into a dedicated database and analyzed statistically. Descriptive statistics were calculated for demographic, radiographic, and clinical variables. Continuous variables are presented as means with ranges or standard deviations when available; categorical variables are presented as counts and percentages. Comparisons of functional scores between 6 and 12 months were performed using Student’s t-test for paired observations when paired follow-up values were available. The normality of distributions was not formally tested because individual patient-level data were not available for all variables at the time of this revision. Missing data were handled by available-case analysis; no imputation was performed. *p* values lower than 0.05 were considered statistically significant. Exact *p* values and confidence intervals could not be calculated from the aggregated retrospective data available for this revision, and this has been acknowledged as a methodological limitation [20].

## 3. Results

**Figure 2:**
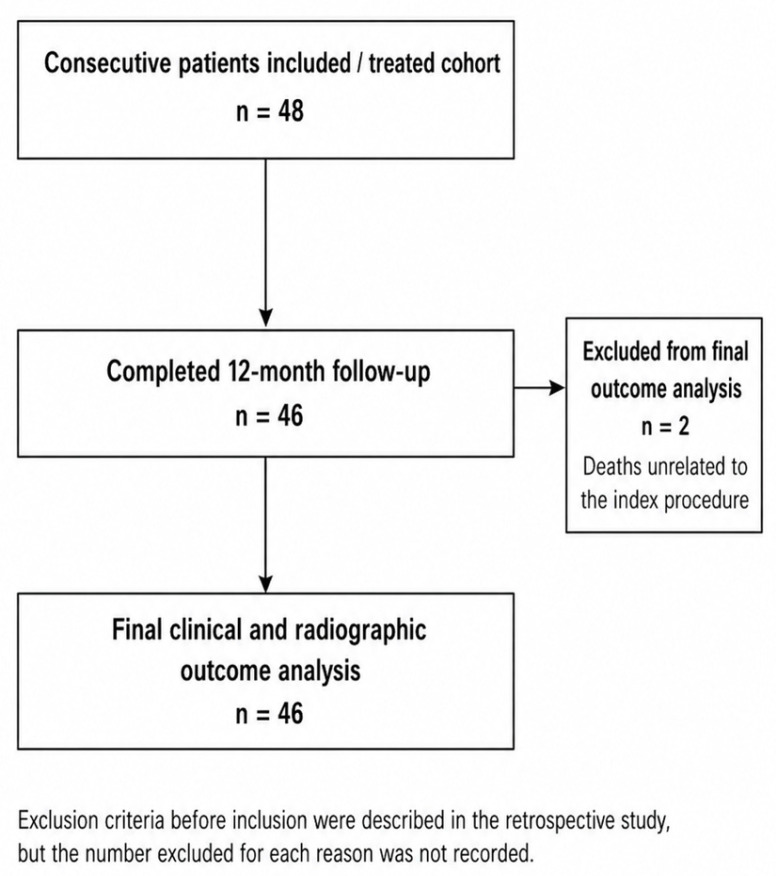
Patient flow diagram.

At a minimum follow-up of 12 months, data were available for 48 patients treated with external fixation for proximal humerus fractures. The mean interval between trauma and surgery was 1.35 days (range, 0–3 days). Mean fluoroscopy exposure was 2.61 mGy, corresponding to an average of 72.5 s, and mean hospital stay was 5.53 days (range, 2–10 days). Five high-volume and six lower-volume shoulder surgeons contributed to patient management from admission to implant removal at a mean of 7 weeks (range, 4–10 weeks). The available patient flow is reported in Figure 2 and Table 2; screening and pre-inclusion exclusion counts were not available in the retrospective dataset.

Forty-six patients completed the 12-month follow-up; two patients died during follow-up from causes unrelated to the index procedure and were excluded from the final clinical and radiographic outcome analysis. Attendance counts at the intermediate 6-week, 3-month, and 6-month visits were not consistently recorded in the retrospective dataset. Minor missing data at intermediate time points were therefore handled by available-case analysis, without imputation. Treatment-related complications are summarized in Table 3.

Functional scores improved over time and are summarized in Table 4. The mean Constant Shoulder Score increased from 62.7 at the 6-month follow-up to 69.3 at 12 months (*p* < 0.05). The mean QuickDASH score improved from 9.4 at 6 months to 8.1 at 12 months (*p* < 0.05), indicating progressive reduction in residual disability. Quality-of-life assessment showed a mean SF-12 Mental Component Score of 44.6 and a mean SF-12 Physical Component Score of 47.2 at follow-up; corresponding 6-month SF-12 component values were not available for tabulation.

Radiographic outcomes are summarized in Table 5. Maintenance of reduction was satisfactory. The mean head-shaft angulation measured on the first postoperative day was 137.2 degrees and remained substantially stable at 135.1 degrees at 12 months, indicating limited loss of correction during healing.

The low rate of revision surgery in our series deserves to be emphasized. In proximal humerus fracture care, revision procedures are especially problematic because they often involve older patients with reduced physiological reserve, poorer soft tissues, and more complex salvage options.

A treatment strategy that limits the need for reoperation may have clinical relevance in this population; however, the present study was not designed as a formal comparative or cost-effectiveness analysis. At 12 months, 46 of 48 patients (95.8%) completed the study. Two patients died during follow-up from causes unrelated to the index procedure and were therefore excluded from the final clinical and radiographic outcome analysis. At intermediate time points (6 weeks, 3 months, and 6 months), minor missing data occurred due to occasional missed visits. However, no systematic pattern of missing data was identified in the available records.

Overall, these findings indicate that external fixation provided enough stability for fracture healing and progressive functional recovery, with a limited need for secondary surgical procedures in this cohort. The study setting, surgical treatment characteristics, and perioperative data are reported in Table 6, providing a detailed overview of the procedural context and early clinical course of the study cohort.

## 4. Discussion

From an epidemiological perspective, our series reflects the typical profile of proximal humerus fractures described in the literature. Their incidence increases with age and is markedly higher in women, largely because osteoporosis reduces bone mineral density and predisposes to fragility fractures. In our cohort, the mean age was 66.3 years in women and 63.3 years in men [18], confirming the close relationship among aging, bone fragility, and the occurrence of PHFs. Most injuries followed low-energy mechanisms, such as domestic falls, whereas a smaller proportion was associated with high-energy trauma in younger individuals.

Management of proximal humerus fractures remains controversial because treatment decisions must balance fracture morphology, biological healing potential, patient-related factors, and the risk of complications associated with each surgical technique. Fracture morphology alone is rarely sufficient to determine the ideal management strategy. The final decision must balance fracture stability, biological healing potential, patient functional demand, comorbidity burden, and risk of complications. This is particularly true in elderly patients, in whom a purely anatomic reconstruction is not always synonymous with the best overall outcome. In this scenario, a technique capable of limiting surgical aggression while preserving reduction may offer substantial advantages.

In our experience, external fixation yielded satisfactory radiographic and clinical outcomes, with progressive improvement in Constant and QuickDASH scores and a limited loss of head-shaft angle over time. One of the most relevant findings is the low rate of revision surgery, which is especially important when treating fragile patients or complex fractures in which implant failure or secondary displacement may carry high clinical costs. The most common complication was pin-tract infection, a known drawback of external fixation, but in our series it was generally manageable with local care and selective pin removal [21,22].

The biological rationale of external fixation deserves particular consideration. Unlike open reduction and internal fixation, this method allows fracture stabilization with limited periosteal stripping and minimal disturbance of the fracture hematoma. Preservation of soft tissues and vascular supply to the humeral head may theoretically reduce the risk of ischemic complications and support fracture healing. In addition, the absence of bulky internal hardware may simplify management if late complications, such as osteonecrosis or secondary collapse, occur.

Other surgical strategies have been widely investigated. Intramedullary nailing has been proposed as an effective solution, especially for selected 2-part surgical-neck fractures, with the advantage of limited soft-tissue exposure and relatively rapid rehabilitation. Percutaneous Kirschner-wire fixation remains a possible option in some younger patients or in selected elderly individuals who are not suitable for major surgery, although stability concerns and secondary displacement can limit its indications. According to Neer [1], more complex 3-part or 4-part fractures have historically been considered candidates for hemiarthroplasty or, more recently, reverse shoulder arthroplasty when reconstruction is deemed unreliable.

At the same time, modern locking plates have significantly expanded the indications for osteosynthesis, particularly in younger or active patients. Angular-stable plate systems provide improved fixation in osteoporotic bone and can achieve excellent reduction when tuberosities and medial support are adequately restored. Nevertheless, the literature reports non-negligible complication and revision rates. Sudkamp et al. [12], in a multicenter study of 187 patients treated with plates and screws, described generally positive recovery but a complication rate of 34% and a revision rate of 19%. Similarly, Hirschmann et al. [14] reported a revision rate of 28% in a series of 57 patients despite satisfactory longer-term function. These findings explain why less-invasive alternatives remain attractive, especially for selected fracture patterns.

External fixation may therefore be interpreted as an evolution of the principle of biologically respectful osteosynthesis. It combines indirect reduction, percutaneous stabilization, and preservation of local vascularity with the possibility of correcting alignment during the early postoperative period. The modular construct may also be especially useful in elderly osteoporotic bone, where screw purchase is less predictable and where shorter operative time can be advantageous. In our cohort, the technique also appeared reproducible across surgeons with different levels of shoulder specialization, supporting the concept of a relatively short learning curve.

Rehabilitation is another central element in the success of this treatment. External fixation provides stability but does not, by itself, guarantee functional recovery. Close patient education regarding frame management, weekly pin care, and adherence to the rehabilitation schedule is essential. Early passive motion helps limit stiffness, whereas delayed yet progressive active motion supports muscle recovery and return to autonomy. Our data suggest that when these aspects are carefully supervised, the technique can deliver acceptable recovery even in an elderly population [12,23].

At the same time, the complication profile of external fixation should be interpreted realistically. Pin-tract infection is common to all external fixation techniques and requires meticulous patient instruction, regular surveillance, and rapid intervention when local inflammation appears. The occurrence of bursitis from pin impingement near the acromion is another specific issue that should be anticipated by both surgeon and rehabilitation team. These complications are generally manageable, but they underline that the technique is not maintenance-free and should ideally be used in settings where structured follow-up can be guaranteed.

Another point of interest is implant removal. Unlike internal fixation devices that may remain in situ or require a second formal operation for removal, the external fixator can usually be removed in an outpatient setting once healing is judged sufficient. This characteristic reduces the burden of retained hardware and may be particularly useful when later conversion to arthroplasty becomes necessary. In this sense, external fixation may preserve future treatment options rather than complicating them.

Finally, the organizational implications of proximal humerus fracture management should be interpreted cautiously. Operative duration, implant cost, need for revision, duration of hospitalization, and intensity of postoperative care all contribute to the overall burden on the healthcare system. However, the present study was not designed as an economic evaluation and did not formally analyze cost-effectiveness, learning curve, or operator-dependent variability. Therefore, comments regarding these aspects should be considered hypothesis-generating only.

The present study nevertheless has important limitations. First, it is a retrospective analysis with a relatively small sample size. Second, there was no control group treated with plates, nails, arthroplasty, or conservative management, and therefore our data cannot establish superiority over competing techniques. Third, the procedures were performed by different surgeons, although the relative consistency of results partially mitigates this concern. Fourth, some follow-up assessments, especially in elderly patients, were not always performed through a fully standardized face-to-face protocol. Finally, longer-term data are lacking, so complications such as late osteonecrosis, secondary glenohumeral osteoarthritis, or delayed mechanical failure may be underrepresented.

Despite these limitations, the findings of this study are clinically relevant because they suggest that external fixation may be considered among the available options for selected proximal humerus fractures, particularly when preservation of biology and avoidance of extensive soft-tissue dissection are priorities. Larger prospective comparative studies are necessary to identify the subgroup of patients who benefit most from this treatment and to clarify its position within the current treatment algorithm for PHF.

## 5. Conclusions

Proximal humerus fractures remain a challenging condition in orthopedic practice and are expected to increase in incidence with the aging population. The heterogeneity of fracture patterns and patient characteristics, together with the absence of universally accepted treatment guidelines, continues to make their management controversial.

In this retrospective single-center case series without a control group, the Galaxy Fixation System was used in a selected group of patients with proximal humerus fractures and was associated with satisfactory radiographic maintenance of reduction, progressive improvement in functional scores, and a low rate of revision surgery at 12-month follow-up. These findings suggest that external fixation may represent a viable treatment option in carefully selected patients who are not ideal candidates for prosthetic replacement.

However, the results of this study should be interpreted in light of its methodological limitations, including the retrospective design, the absence of a control group, and the relatively limited sample size. Therefore, no definitive conclusions can be drawn regarding the comparative effectiveness of this technique, its generalizability to other clinical settings, or its reproducibility across different levels of surgical experience. Similarly, aspects such as learning curve and cost-effectiveness were not formally evaluated and cannot be inferred from the present data.

The minimally invasive nature of the technique and the preservation of fracture biology may represent potential advantages, particularly in osteoporotic bone, but these considerations remain observational. No formal analysis of learning curve, reproducibility, transferability, or cost-effectiveness was performed. Careful patient selection, appropriate surgical expertise, and strict postoperative management remain essential to optimize outcomes and minimize complications.

Further prospective, comparative studies with standardized outcome measures are required to better define the role of external fixation in the treatment algorithm of proximal humerus fractures.

**Figure 3:**
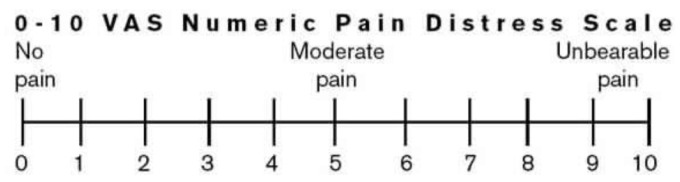
VAS pain distress scale.

Clinical and radiographic follow-up was scheduled at 6 weeks and at 3, 6, and 12 months postoperatively. Functional outcome was assessed using the Constant Score and QuickDASH at 6 and 12 months. Pain intensity was evaluated using the Visual Analogue Scale (VAS) (Figure 3). Quality of life was investigated by means of the SF-12 questionnaire, from which both the Mental Component Score (MCS) and Physical Component Score (PCS) were extracted.

## Figures and Tables

**Table 1:** Baseline demographic and fracture-related characteristics.

Variable	Value
Age, mean (range)	68 years (41–85)
Sex	36 female patients (75%); 12 male patients (25%)
Mean age by sex	Male: 63.3 years; female: 66.3 years
Mechanism of injury	Predominantly low-energy trauma/domestic falls in older patients, with a smaller proportion of high-energy trauma in younger patients. Exact counts were not recorded.
Fracture classification—Neer/fracture pattern	Fractures were classified according to Neer. Eligible/treatment patterns included 2-part, 3-part and selected 4-part proximal humeral fractures with at least two-thirds of intact metaphyseal bone stock. Exact distribution by Neer category was not available for reliable tabulation.
AO/OTA classification	Fractures were classified according to AO/OTA by two senior orthopedic surgeons. Exact AO/OTA distribution was not available for reliable tabulation.
Hertel classification	Fractures were classified according to Hertel by two senior orthopedic surgeons. Exact Hertel distribution was not available for reliable tabulation.
Affected side/laterality	The manuscript refers to the injured arm/affected shoulder; right/left laterality distribution was not available in the retrospective dataset.
Dominant side involved	Not available in the retrospective dataset.
Time from trauma to surgery	1.35 days (range, 0–3)
Osteoporosis/bone quality	Osteoporotic bone and bone quality are discussed as relevant clinical factors; patient-level osteoporosis counts were not available.
Relevant comorbidities	Severe systemic illness, major comorbidities, active cancer/chemotherapy, chronic steroid therapy, sepsis and infection were exclusion criteria; cohort-level comorbidity counts were not available.
ASA score	ASA 4 status was excluded; ASA I-III patients were potentially eligible. Exact ASA distribution was not available.

Values are reported exactly as available in the retrospective manuscript dataset. Variables not available for reliable tabulation are explicitly indicated as not available.

**Table 2:** Participant flow and final analysis cohort.

Stage	N/Value	Explanation
Patients assessed for eligibility	Not available	The total number of patients screened before application of eligibility criteria was not recorded in the retrospective dataset.
Consecutive patients included/treated cohort	48	Forty-eight consecutive patients with proximal humerus fractures underwent surgical treatment with external fixation.
Excluded before inclusion	Not available	Exclusion criteria are described, but the number excluded for each criterion was not recorded.
Treated with external fixation/Galaxy Fixation System	48	All included patients were treated with external fixation.
6-week follow-up	Not available	Scheduled follow-up visit; attendance count was not consistently recorded.
3-month follow-up	Not available	Scheduled follow-up visit; attendance count was not consistently recorded.
6-month follow-up	Not available	Scheduled follow-up visit; attendance count was not consistently recorded.
12-month follow-up completed	46	Forty-six patients completed the minimum 12-month follow-up.
Lost/excluded from final outcome analysis	2	Two patients died during follow-up from causes unrelated to the index procedure.
Final outcome analysis	46	Final clinical and radiographic analysis was performed on 46 patients.

**Table 3:** Treatment-related complications.

Complication Type	Number of Cases	Timing	Treatment Required	Final Outcome
Pin-tract infection	5 (10%)	2–6 weeks postoperatively	Oral antibiotics; pin removal in 4 cases	Resolved without sequelae
Pin migration	4 (8.3%)	3–5 weeks postoperatively	Pin removal	No functional impairment
Algodystrophic syndrome (CRPS)	1 (2%)	Approximately 8 weeks postoperatively	Physiotherapy and analgesics	Partial recovery
Avascular necrosis	1 (2%)	Approximately 6 months postoperatively	Revision surgery with arthroplasty	Improved after revision

**Table 4:** Main clinical outcomes.

Outcome	6 Months	12 Months	*p* Value
Constant Shoulder Score	62.7	69.3	<0.05
QuickDASH	9.4	8.1	<0.05
SF-12 Mental Component Score (MCS)	Not available	44.6	Not applicable
SF-12 Physical Component Score (PCS)	Not available	47.2	Not applicable

Functional outcomes improved between 6 and 12 months. SF-12 component scores were reported at follow-up without corresponding 6-month component values in the available dataset.

**Table 5:** Radiographic outcome.

Parameter	Postoperative/First Postoperative Day	12 Months
Head-shaft angle	137.2 degrees	135.1 degrees

**Table 6:** Study setting, surgical treatment and perioperative data.

Variable	Value
Study design	Retrospective single-center case series without a control group
Setting	Orthopaedic and Traumatology Unit, Versilia Hospital, Viareggio, Italy
Study period	November 2017 to February 2022
Preoperative imaging	Anteroposterior and lateral trauma-series shoulder radiographs; computed tomography when radiographs were insufficient for fracture morphology, tuberosity displacement or surgical planning.
Surgeons	Eleven surgeons; Section 3 describes five high-volume and six lower-volume shoulder surgeons.
Surgical indication	2-part, 3-part and selected 4-part proximal humeral fractures with at least two-thirds of intact metaphyseal bone stock.
Standard construct	Six fully threaded 2.5 mm pins connected in a lambda configuration.
Antibiotic prophylaxis	Ceftriaxone 2 g intravenously; vancomycin 1 g in allergic patients.
Mean fluoroscopy exposure	2.61 mGy
Mean fluoroscopy time	72.5 s
Mean hospital stay	5.53 days (range, 2–10)
Galaxy system removal	43 days (SD ± 3.12) in postoperative care; Results also describe implant removal at a mean of 7 weeks (range, 4–10 weeks).
Follow-up schedule	6 weeks, 3 months, 6 months and 12 months postoperatively.

## Data Availability

The original contributions presented in this study are included in the article and/or Supplementary Materials. Further inquiries can be directed to the corresponding author.

## Supplementary Materials

The following supporting information can be downloaded at: https://www.mdpi.com/article/10.3390/jcm15093432/s1, Table S1: represented patient sex division and follow up classification due to shoulder score.
