# Supplementary material for: External Fixation in the Treatment of Proximal Humeral Fractures: A Retrospective Single-Center Case Series
Source: J Clin Med. 2026 Apr 30;15(9):3432. doi: 10.3390/jcm15093432 (PMC13163877; doi:10.3390/jcm15093432)
Supplement: Supplementary file 1 [file jcm-15-03432-s001.zip › jcm-4212985-supplementary.pdf]

Journal of
Clinical Medicine

MDPI

Table S1. Represented patient sex division and follow up classification due to shoulder score.

Patients	N (48)	Age (68 years)
Male	12	63.3
Female	36	66.3
